# Supplementary material for: Spatial Profile of Tumor Microenvironment in PD-L1-Negative and PD-L1-Positive Triple-Negative Breast Cancer
Source: Int J Mol Sci. 2023 Jan 11;24(2):1433. doi: 10.3390/ijms24021433 (PMC9862087; doi:10.3390/ijms24021433)
Supplement: Supplementary file 1 [file ijms-24-01433-s001.zip › ijms-2096454-supplementary/Table S1.pdf]

Table S1. Morphological annotation and marker genes of clusters in breast cancer tissues

Patient	Morphological annotation of clusters / Marker genes									
	Cluster 1	Cluster 2	Cluster 3	Cluster 4	Cluster 5	Cluster 6	Cluster 7	Cluster 8	Cluster 9	Cluster 10
1	Invasive cancer / MUCL1 SCGB3A1 SCGB2B2 CD24	Invasive cancer, adipose tissue and connective tissue / UPK1A HPD MUC6 KRT7	Invasive cancer, adipose tissue and connective tissue / CD36 SCGB3A1 TXNIP PLAAT4	Connective tissue / COL1A2 CRIP1 S100A8 GLUL	Adipose tissue / SERPINE1 PLPP1	Invasive cancer and connective tissue / STAC2 HPGD S100A7 CXCL13	Immune infiltrate / CD74 CST3 HLA-DRA C3	Connective tissue / IGHG3 CD74 HLA-DRA		
2	Invasive cancer, immune infiltrate and connective tissue / CXCL8 CXCL10 CHI3L2 KRT7	Invasive cancer, immune infiltrate and connective tissue / CD44 VEGFA CD24 CDH1	Invasive cancer, necrosis, immune infiltrate and connective tissue / NDRG1 COL6A1	Invasive cancer and connective tissue / CCL21 JCHAIN SFRP4 COL1A2	Invasive cancer and connective tissue	Invasive cancer and connective tissue / SAA2 SERPINA3 SRSF2 IGHA1	Invasive cancer and immune infiltrate / CD74 LYZ HLA-DRA COL4A1	Invasive cancer and immune infiltrate / HLA-DPA1 HLA-DPB1 CD163 LAPTM5	Invasive cancer, necrosis and connective tissue	
3	Invasive cancer, immune infiltrate and connective tissue / PROM1 CD151 KLK6 DSC3 KRT7 EPCAM	Invasive cancer, immune infiltrate and connective tissue / MMP7 KRT14 PTMS CRABP1 CD24	Invasive cancer, immune infiltrate and connective tissue / CD74 TIMP1 HLA-DRA IGLV3-1	Invasive cancer, immune infiltrate and connective tissue / CXCL13 HLA-DPB1 HLA-DRA IGHA1 PTPRC	Invasive cancer, immune infiltrate and connective tissue / S100A10 SDC1 CRYAB MGP	Invasive cancer, immune infiltrate and connective tissue / MLF2 LAD1 SPIB CHI3L2	Immune infiltrate, single tumor cells / LTF S100A9 S100A8 CSTB	Immune infiltrate / COL11A1 HLA-DPA1 HLA-DRA CDH11	Immune infiltrate and normal glands / COL6A1 COL3A1 FN1 COL5A1	Vessels
4	Invasive cancer, immune	Invasive cancer, immune infiltrate and	Immune infiltrate, single	Immune infiltrate,	Connective tissue / IL6 CD34	Connective tissue / CXCL13	Vessels / COL1A1 DCN			

	infiltrate and connective tissue / CD79B IGHA1 GZMB CD28 CD22	connective tissue / KRT81 KRT16 KRT7 IL32	tumor cells / IGHG1 SERPINA1 IGLV3-1 CXCL14	single tumor cells / KRT7 LTF CD44 CD24	TGFBR2 CXCL12	HLA-DRA CD74 CD52	C3 CCL2			
5	Connective tissue / DCN SELENOP IGHA1 CXCL12	Tertiary lymphoid structures / CD19 CD22 CD72 CD79B	Connective tissue, immune infiltrate and normal glands / PIGR EEF2 APOD CXCL2 SFRP1	Connective tissue and normal glands / C1S C3 CCDC80 DCN	Invasive cancer / KRT16 KRT6B KRT81 MMP1	Connective tissue and single tumor cells / CD28 CD37 CD3D CD3E PTPRC	Invasive cancer (tumor-stroma border) / CXCL10 HLA-DRA COL1A1 CXCL11 CD74	Invasive cancer inside tertiary lymphoid structures / IL1R2 IL22RA2 CHI3L2 KRT15	Adipose tissue and connective tissue / CD163 CD63 COL1A1 COL1A2	
6	Invasive cancer, immune infiltrate, connective tissue, normal glands and necrosis / COL1A2 SPARC FN1 COL6A3	Invasive cancer, immune infiltrate, connective tissue and necrosis / KLK5 KRT15 CALML5 CX3CL1	Connective tissue and normal glands / FOSB FOS	Connective tissue, invasive carcinoma, tertiary lymphoid structures and normal glands / IGLC1 IGHG1 IGHG2 IGKC	Connective tissue, invasive carcinoma and normal glands / KRT8 KRT7 CD24 PROM1	Connective tissue, invasive carcinoma and tertiary lymphoid structures / MMP7 PIGR S100A9 ALCAM CD24				
