# Supplementary material for: Spatial Profile of Tumor Microenvironment in PD-L1-Negative and PD-L1-Positive Triple-Negative Breast Cancer
Source: Int J Mol Sci. 2023 Jan 11;24(2):1433. doi: 10.3390/ijms24021433 (PMC9862087; doi:10.3390/ijms24021433)
Supplement: Supplementary file 1 [file ijms-24-01433-s001.zip › ijms-2096454-supplementary/Table S2.pdf]

Table S2. GO terms in tissue clusters of PD-L1-negative and PD-L1-positive triple-negative breast cancer

Cluster number	PD-L1-negative cases			PD-L1-positive cases		
	1	2	3	4	5	6
1	cell migration involved in sprouting angiogenesis (GO:0002042) p = 0.024 negative regulation of cell cycle (GO:0045786) p = 0.047 positive regulation of apoptotic process (GO:0043065) p = 0.047		neutrophil activation involved in immune response (GO:0002283) p = 3.62E-4		extracellular matrix organization (GO:0030198) p = 3.54E-7 positive regulation of cell motility (GO:2000147) p = 1.63E-4	
2	-		negative regulation of angiogenesis (GO:0016525) p = 2.61E-5 regulation of cell migration (GO:0030334) p = 0.0012	regulation of cell population proliferation (GO:0042127) p = 0.024 negative regulation of cell motility (GO:2000146) p = 0.024	B cell activation (GO:0042113) p = 2.37E-7 lymphocyte differentiation (GO:0030098) p = 2.73E-6 T-helper cell lineage commitment (GO:0002295) p = 0.009	
3	antigen processing and presentation of exogenous peptide antigen (GO:0002478) p = 1.44E-10 positive regulation of cytokine production (GO:0001819) p = 1.399E-7		cytokine-mediated signaling pathway (GO:0019221) p = 4.54E-10 negative regulation of programmed cell death (GO:0043069) p = 5.20E-4 antigen processing and presentation of peptide		extracellular matrix organization (GO:0030198) p = 3.16E-4 regulation of insulin-like growth factor receptor signaling pathway (GO:0043567) p = 0.0024	cellular response to type I interferon (GO:0071357) p = 3.15E-10 positive regulation of

			antigen via MHC class I (GO:0002474) p = 0.011		positive regulation of cell motility (GO:2000147) p = 0.004	fibroblast proliferation (GO:0048146) p = 7.29E-4
4	extracellular matrix organization (GO:0030198) p = 1.31E-29 negative regulation of cell migration (GO:0030336) p = 4.12E-4	extracellular matrix organization (GO:0030198) p = 1.95E-26 negative regulation of angiogenesis (GO:0016525) p = 6.30E-8 cytokine-mediated signaling pathway (GO:0019221) p = 4.846E-4 antigen processing and presentation of exogenous peptide antigen via MHC class II (GO:0019886) p = 4.03E-4	extracellular structure organization (GO:0043062) p = 0.0033 regulation of cell migration (GO:0030334) p = 0.013	defense response to virus (GO:0051607) p = 9.63E-6	positive regulation of cell population proliferation (GO:0008284) p = 2.23E-6 negative regulation of apoptotic process (GO:0043066) p = 8.15E-4 negative regulation of epithelial to mesenchymal transition (GO:0010719) p = 0.0016 positive regulation of cell motility (GO:2000147) p = 0.0018	cytokine-mediated signaling pathway (GO:0019221) p = 3.958E-11 positive regulation of T cell activation (GO:0050870) p = 3.400E-5 interferon-gamma-mediated signaling pathway (GO:0060333) p = 0.0013 regulation of T-helper 1 cell differentiation (GO:0045625)

						p = 0.0101
5			extracellular matrix organization (GO:0030198) p = 4.624E-22 neutrophil activation involved in immune response (GO:0002283) p = 8.09E-10 cytokine-mediated signaling pathway (GO:0019221) p = 1.743E-7 antigen processing and presentation of exogenous peptide antigen (GO:0002478) p = 4.448E-4	extracellular structure organization (GO:0043062) p = 1.03E-16 cytokine-mediated signaling pathway (GO:0019221) p = 0.0052 neutrophil activation involved in immune response (GO:0002283) p = 0.025 cellular response to transforming growth factor beta stimulus (GO:0071560) p = 0.0390	cellular response to type I interferon (GO:0071357) p = 3.70E-4 negative regulation of transforming growth factor beta production (GO:0071635) p = 0.0020 regulation of cell population proliferation (GO:0042127) p = 0.0093	extracellular matrix organization (GO:0030198) p = 4.65E-11 cellular response to cytokine stimulus (GO:0071345) p = 0.0060
6	fatty acid beta-oxidation (GO:0006635) p = 5.31E-4		extracellular matrix organization (GO:0030198) p = 2.92E-8 neutrophil activation involved in immune response (GO:0002283) p = 5.32E-7 negative regulation of cell migration (GO:0030336) p = 5.32E-7 cytokine-mediated signaling pathway (GO:0019221) p = 3.76E-5	extracellular matrix organization (GO:0030198) p = 1.874E-9	extracellular matrix organization (GO:0030198) p = 3.03E-12 cellular response to cytokine stimulus (GO:0071345) p = 6.760E-11 antigen receptor-mediated signaling pathway (GO:0050851) p = 2.788E-6	epithelium development (GO:0060429) p = 0.011 negative regulation of programmed cell death (GO:0043069) p = 0.035

7	fatty acid beta-oxidation (GO:0006635) p = 7.634E-6		cytokine-mediated signaling pathway (GO:0019221) p = 9.42E-7 negative regulation of intrinsic apoptotic signaling pathway (GO:2001243) p = 4.011E-6 neutrophil activation involved in immune response (GO:0002283) p = 4.011E-6	extracellular matrix organization (GO:0030198) p = 2.42E-15 positive regulation of cytokine production (GO:0001819) p = 4.38E-7 regulation of angiogenesis (GO:0045765) p = 1.267E-5	extracellular matrix organization (GO:0030198) p = 2.44E-15 neutrophil mediated immunity (GO:0002446) p = 6.69E-9 positive regulation of cell migration (GO:0030335) p = 9.175E-5	
8			negative regulation of RNA polymerase II regulatory region sequence-specific DNA binding (GO:1903026) p = 0.0151		positive regulation of wound healing (GO:0090303) p = 0.0225 regulation of cell migration (GO:0030334) p = 0.0491	
9			regulation of immune effector process (GO:0002697) p = 0.0030 positive regulation of immune response (GO:0050778) p = 0.0042 adipose tissue development (GO:0060612) p = 0.0044		extracellular matrix organization (GO:0030198) p = 1.269E-6 endothelium development (GO:0003158) p = 0.00270	
